# Vertebral body collapse after spine stereotactic body radiation therapy: a single-center institutional experience

**DOI:** 10.2478/raon-2024-0033

**Published:** 2024-06-12

**Authors:** Arsh Issany, Austin J Iovoli, Richard Wang, Rohil Shekher, Sung Jun Ma, Victor Goulenko, Fatemeh Fekrmandi, Dheerendra Prasad

**Affiliations:** Jacobs School of Medicine and Biomedical Sciences, University at Buffalo, New York, USA; Department of Radiation Medicine, Roswell Park Comprehensive Cancer Center, New York, USA; Kirk Kerkorian School of Medicine, University of Nevada, Las Vegas, USA

**Keywords:** spine metastasis, stereotactic body radiation therapy, vertebral compression fracture, kyphoplasty, spinal instability

## Abstract

**Background:**

Spine stereotactic body radiation therapy (SBRT) for the treatment of metastatic disease is increasingly utilized owing to improved pain and local control over conventional regimens. Vertebral body collapse (VBC) is an important toxicity following spine SBRT. We investigated our institutional experience with spine SBRT as it relates to VBC and spinal instability neoplastic score (SINS).

**Patients and methods:**

Records of 83 patients with 100 spinal lesions treated with SBRT between 2007 and 2022 were reviewed. Clinical information was abstracted from the medical record. The primary endpoint was post-treatment VBC. Logistic univariate analysis was performed to identify clinical factors associated with VBC.

**Results:**

Median dose and number of fractions used was 24 Gy and 3 fractions, respectively. There were 10 spine segments that developed VBC (10%) after spine SBRT. Median time to VBC was 2.4 months. Of the 11 spine segments that underwent kyphoplasty prior to SBRT, none developed subsequent VBC. No factors were associated with VBC on univariate analysis.

**Conclusions:**

The rate of vertebral body collapse following spine SBRT is low. Prophylactic kyphoplasty may provide protection against VBC and should be considered for patients at high risk for fracture.

## Introduction

About a third of cancer patients will develop bone metastases during the course of their disease.^1^ The most common site of bone metastasis is the spine, which can often present with back pain, vertebral body collapse (VBC), radiculopathy, and epidural spinal cord compression.^2^ Compression of the spinal cord has the potential to cause serious harm with symptoms ranging from pain to paralysis.^2^ Optimal management of spine metastases involves multidisciplinary collaboration between surgeons, medical oncologists, pain specialists, and radiation oncology. For patients who are not candidates for immediate neurosurgical intervention, management often involves palliative radiotherapy with the goal of providing symptomatic relief of pain and preventing further progression of disease. With technological advancements in the delivery of radiotherapy, stereotactic body radiation therapy (SBRT) has emerged as an effective technique to safely treat spinal metastases with high doses of radiation while sparing surrounding healthy tissue.^3^

Delivery of spine SBRT involves precise treatment planning and patient setup utilizing computed topography image verification to ensure the radiation is delivered conformally to the target. The advantages of treating malignant spine metastases with SBRT is controversial. A recent meta-analysis showed the overall pain response may be similar compared to conventional external beam radiotherapy (cEBRT), but more patients had complete pain alleviation with SBRT.^4^ Other studies have shown advantages of SBRT compared to cEBRT such as improved local control and pain relief.^5,6,7^ With advances in systemic therapies improving survival for many types of malignancies, local control of all metastatic disease has become increasingly important. Several drawbacks to spine SBRT, however, are increased risks of pain flare and radiation induced VBC. Current literature suggests the rate of VBC is between 4% and 39% for patients with metastatic disease undergoing spine SBRT.^8,9,10,11,12,13^ Chronic pain and kyphotic deformity caused by VBC may lead to depression, impaired mobility, and reduced quality of life.^14^ One study also found no clinically relevant differences between conventional radiotherapy and SBRT at 12 weeks for global quality of life, physical functioning, emotional functioning, functional interference, and psychosocial aspects.^15^ This necessitates further exploration into the side effects of SBRT.

Multiple risk factors for VBC following spine SBRT have been identified, which include vertebral body involvement, kyphotic/scoliotic spine deformity, lytic tumor, lung and hepatocellular histology, and single-fraction SBRT to a dose of 20 Gy or higher.^9,16^ In developing a tool to predict the risk of VBC after spine SBRT, epidural tumor extension, lumbar location, gross tumor volume, and spinal instability neoplastic score (SINS) of more than 6 were associated with increased risk of fracture.^17^ While risk factors and predictive models are helpful in identifying patients at high risk of VBC, it remains unclear how to best reduce fracture incidence while also providing effective palliation.

Kyphoplasty is a minimally invasive procedure used in the management of VBC that uses an inflatable balloon to restore bone height then inject bone cement into the vertebral body.^18^ This has been shown to be safe and effective for controlling pain in patients with spine metastases.^19,20^ Few studies have investigated the effect of prophylactic kyphoplasty prior to spine SBRT on reducing the risk of VBC. The purpose of the present study is to expand on the published experience of spine SBRT and review our single-institution outcomes of spine SBRT with and without prophylactic kyphoplasty as it relates to SINS and VBC.

## Patients and methods

### Study population

The patient cohort was derived from all patients who received spine SBRT at a single institution between March 2007 and May 2022. Patients with tumors on the spinal cord or dura were excluded. The primary endpoint was development of VBC following completion of spine SBRT, defined as a new VBC or progression of an existing VBC. Data were collected under a protocol (BDR 157322) approved by the Institutional Review Board at Roswell Park Comprehensive Cancer Center. The Strengthening the Reporting of Observational Studies in Epidemiology (STROBE) reporting guideline was followed.

### Patient data and treatment

Pertinent clinicopathologic data were abstracted from the electronic medical record for patients treated with spine SBRT. Clinically relevant variables included gender, race, age, Karnofsky Performance Status (KPS), primary malignancy, SINS, kyphoplasty performed, paraspinal extension, treatment dose, and treatment fractionation. SINS was calculated for each vertebral segment treated per published criteria using tumor location, pain, bone lesion type, radiographic spinal alignment, VBC, and posterolateral involvement of spinal elements.^21^ Pre-treatment and post-treatment computed tomography (CT) and magnetic resonance imaging (MRI) of the spine was reviewed to obtain pertinent data. Data was collected from the radiation consultation visit prior to the delivery of SBRT and at the time of first imaging follow up after treatment completion. Dose prescribed was at the discretion of the treating radiation oncologist based on pertinent clinicopathologic factors. Institutional protocols outlining dose constraints to surrounding tissue were followed. There was no maximum dose constraint in the target as long as all dose constraints were met. Eclipse (Varian Medical Systems, Palo Alto, CA, USA) was used for the generation and evaluation of radiation treatment plans. We contoured clinical target volume (CTV) and planning target volume (PTV) according to Consensus Contouring Guidelines.^22^ SBRT was delivered with a Varian TrueBeam utilizing online cone beam CT imaging, high definition multileaf collimator, and a 6 degrees of freedom couch.

### Statistics

Univariate logistic regression using the log-rank method was used to identify factors associated with development of VBC. All p-values were two-sided and variables with p < 0.05 were considered statistically significant. Statistical Analysis was conducted using R (version 4.2.0, R Project for Statistical Computing, Vienna, Austria).

### Ethical statement

The authors are accountable for all aspects of the work in ensuring that questions related to the accuracy or integrity of any part of the work are appropriately investigated and resolved. The study was conducted in accordance with the Declaration of Helsinki (as revised in 2013). The study was approved by the institutional review board of Roswell Park Comprehensive Cancer Center (BDR 157322).

## Results

A total of 83 patients with 100 treated spine segments were included for analysis. There were 10 patients with simultaneously treated synchronous metastases and 7 patients with metachronous metastases. Baseline patient characteristics and treatment details are described in Table 1. The median age was 68 years (interquartile range [IQR], 59−74) and 51% of patients were male. Median follow up time was 12.1 months (IQR, 5.0−25.5). The most common primary tumor histology treated was lung (29%), followed by renal (24%) and prostate (12%). Median dose and number of fractions used was 24 Gy and 3 fractions, respectively. Categories with (n = 100) are lesion-level variables and categories with (n = 83) are patient variables. The 100 lesions were assumed independent events for patients with multiple lesions. SINS for each spine segment prior to SBRT are summarized in Table 2.

**TABLE 1.** Baseline patient characteristics and treatment information

	**VBC (N = 10)**	**%**	**No VBC (N = 90)**	**%**	**Total (N = 100)**	**%**
Median follow up, month (IQR) (n = 100)	10.9	3.9–18.6	12.5	5.1–27.2	12.1	5.0–25.5
Sex (n = 83)*						
Male	5	56%	37	50%	42	51%
Female	4	44%	37	50%	41	49%
Median age, year (IQR) (n = 83)*	67	63–70	69	59–75	68	59–74
Race (n = 83)						
White	8	89%	69	93%	77	93%
Black	2	22%	1	1%	3	4%
Other/unknown	0	0%	3	4%	3	4%
KPS (n = 83)*						
≥ 80	9	90%	70	95%	79	95%
< 80	1	10%	3	4%	4	5%
Primary tumor (n = 100)**						
Lung	4	40%	25	28%	29	29%
Renal	1	10%	23	26%	24	24%
Breast	1	10%	8	9%	9	9%
Prostate	2	20%	10	11%	12	12%
Melanoma	0	0%	2	2%	2	2%
Other	2	20%	22	24%	24	24%
Spine Level (n = 100)**						
Cervical	1	10%	14	16%	15	15%
Thoracic	6	60%	58	64%	64	64%
Lumbosacral	3	30%	18	20%	21	21%
Kyphoplasty pre-SBRT (n = 100)**						
Yes	0	0%	11	12%	11	11%
No	10	100%	79	88%	89	89%
Paraspinal extension (n = 100)**						
Yes	4	40%	36	40%	40	40%
No	6	60%	54	60%	60	60%
Total dose (Gy)/fractions (n = 100)**						
12−17/1	1	10%	12	13%	13	13%
10−24/2	0	0%	2	2%	2	2%
15−30/3	8	80%	61	68%	69	69%
20-30/4–5	1	10%	15	17%	16	16%
Dose (Gy) per fraction (n = 100)**						
< 8	4	40%	31	34%	35	35%
8−12	5	50%	50	56%	55	55%
13−17	1	10%	9	10%	10	10%

IQR = interquartile range; KPS = Karnofsky performance status; SBRT = stereotactic body radiation therapy; VBC = vertebral body collapse

* Categories with designation (n = 100) are lesion-level variables;

** Categories with (n = 83) are patient variables

**TABLE 2.** Pre-treatment patient spinal instability neoplastic score (SINS) outcomes

	**VBC (n=10)**	**%**	**No VBC (n = 90)**	**%**	**Total (n = 100)**	**%**
Location						
Junctional (O-C2; C7−T2; T11−L1; L5−S1)	3	30%	32	36%	35	35%
Mobile spine (C3−6; L2−4)	3	30%	18	20%	21	21%
Semirigid (T3−10)	4	40%	40	44%	44	44%
Rigid (S2−5)	0	0%	0	0%	0	0%
Mechanical pain						
Yes	5	50%	49	54%	54	54%
No	3	30%	30	33%	33	33%
Pain-free lesion	2	20%	11	12%	13	13%
Bone lesion						
Lytic	8	80%	71	79%	79	79%
Mixed (lytic/blastic)	1	10%	9	10%	10	10%
Blastic	1	10%	10	11%	11	11%
Radiographic spinal alignment						
Subluxation/translation present	0	0%	8	9%	8	8%
Deformity (kyphosis/scoliosis)	2	20%	17	19%	19	19%
Normal	8	80%	65	72%	73	73%
Vertebral body collapse (p = 0.040)						
> 50% collapse	0	0%	9	10%	9	9%
< 50% collapse	1	10%	8	9%	9	9%
No collapse with > 50% body involved	1	10%	38	42%	39	39%
None of the above	8	80%	35	39%	43	43%
Posterolateral involvement						
Bilateral	0	0%	5	6%	5	5%
Unilateral	6	60%	33	37%	39	39%
None of the above	4	40%	52	58%	56	56%
SINS classification						
Stable	4	40%	22	24%	26	26%
Potentially instability	6	60%	63	70%	69	69%
Unstable	0	0%	5	6%	5	5%

VBC = vertebral body collapse

Following SBRT there were 10 spine segments that developed VBC (10%), 9 which were de novo VBC and 1 that was progression of a prior VBC. Median time to VBC was 2.4 months (IQR, 0.9−4.0). Of the 11 spine segments that underwent kyphoplasty prior to SBRT, none developed subsequent VBC. No clinical or SINS factors were associated with VBC upon univariate analysis.

## Discussion

This study reviewed a large single-institutional experience with spine SBRT through evaluation of VBC and SINS. As implementation of spine SBRT into practice continues to evolve, there is greater need for tools to identify patients at highest risk of adverse events such as VBC. We report the risk of VBC to be 10%, which agrees with other studies that found the risk to range from 4% to 39%.^8,9,10,11,12,13^ The wide range of published VBC rates likely owes to differences in treatment technique and patient selection. Unlike previous reports, our study was unable to identify additional clinical or SINS factors associated with VBC. A systematic review of studies examining risk of VBC post-SBRT and reporting risk factors identified lytic disease, baseline VBC prior to SBRT, higher dose per fraction SBRT, spinal deformity, older age, and more than 40% to 50% of vertebral body involved by tumor to be the most frequent factors associated with VBC on Multivariable analysis.^23^

**TABLE 3.** Logistic univariate analysis of factors associated with vertebral body collapse

	**Univariate analysis p-value**
Gender	0.60
Age (≥ 68 vs. < 67)	0.89
KPS (≥ 80 *vs*. < 80)	0.38
Spine level (cervical *v.s* thoracic)	0.74
Spine Level (cervical *vs*. lumbosacral)	0.48
Spine Level (thoracic *vs*. lumbosacral)	0.53
Paraspinal extension	1.00
Dose per fraction (< 9 Gy v.s ≥ 9 Gy)	0.43
Location (rigid/semi-rigid *vs* mobile/junctional spine)	0.79
Mechanical pain	0.79
Lytic *vs* non-lytic bone lesion	0.93
Spinal alignment (normal *vs.* kyphosis/scoliosis)	0.96
Posterolateral involvement	0.29

CI = confidence interval; HR = Hazard ratio; KPS = Karnofsky performance status; SBRT = stereotactic body radiation therapy

Management of a radiation induced VBC can be challenging and may require surgical intervention. In a review of patients developing VBC after spine SBRT, they found that 32% of patients needed a salvage spinal reconstruction procedure, consisting primarily of percutaneous cement augmentation procedures in 77% of patients while the remaining patients required open spinal reconstructive surgery.^24^ Method of salvage intervention is institutionally dependent and will vary based on resources available, clinical factors, and patient performance status. While spinal instrumentation may provide greater stability than cement augmentation procedures such as kyphoplasty, these are more invasive procedures and typically result in more post-operative pain.

A key finding from our study is no post-treatment VBC occurred in patients that underwent prophylactic kyphoplasty prior to SBRT. While kyphoplasty prior to spine SBRT has previously been shown to be safe and effective in small series, neither reported rates of subsequent VBC.^25,26^ In agreement with these findings, another study found the incidence of VBC to be lower in patients that underwent surgical intervention or vertebroplasty prior to SBRT.^17^ The optimal timing and patient selection for kyphoplasty in those undergoing spine SBRT still remains under investigation. By utilizing previously identified risk factors, patients at high risk of fracture should be considered for kyphoplasty to protect them from complications prior to ablative therapy with SBRT.

### Limitations

This study has multiple limitations. As with any retrospective study, there may be loss of data and miscoding during abstraction from the medical record. Additionally, our cohort was limited by the number of patients included. The sample size and low number of VBC events may not have been sufficient to confirm previously identified risk factors for VBC with statistical significance. Another limitation is the heterogeneity of patient clinical factors and years treated, which resulted in variation in how patients were approached with SBRT and different follow-up imaging protocols. Despite these limitations, this study presents valuable data demonstrating low rates of VBC following spine SBRT and the potential protective effects of prophylactic kyphoplasty on further reducing this rate in appropriately selected patients. Furthermore, some patients didn’t receive ablative dose to vertebra, only palliative dose was delivered. One patient received only 2x5 Gy and there are few with 5x4 Gy fractionation.

## Conclusions

The rate of vertebral body collapse following spine SBRT is low. Prophylactic kyphoplasty may provide protection against VBC and should be considered for patients at high risk for fracture.

## Acknowledgements

### Funding support

This project is supported in pa by funding from the National Cancer Institute of the National Institutes of Health under Award number: R25CA181003. The funding sources had no role in the preparation of this manuscript.

### Author contributions

Arsh Issany: Data Curation, Investigation, Formal Analysis, Writing - Original Draft. Austin J. Iovoli: Supervision, Writing – Review & Editing. Richard Wang: Data Curation, Writing – Review & Editing. Rohil Shekher: Methodology, Writing – Review & Editing. Sung Jun Ma: Methodology, Supervision, Writing – Review & Editing. Victor Goulenko: Writing – Review & Editing. Fatemeh Fekrmandi: Writing – Review & Editing. Dheerendra Prasad: Conceptualization, Validation, Supervision, Writing – Review & Editing.

### Data sharing

Research data are stored in an institutional repository and will be shared upon request to the corresponding author.
